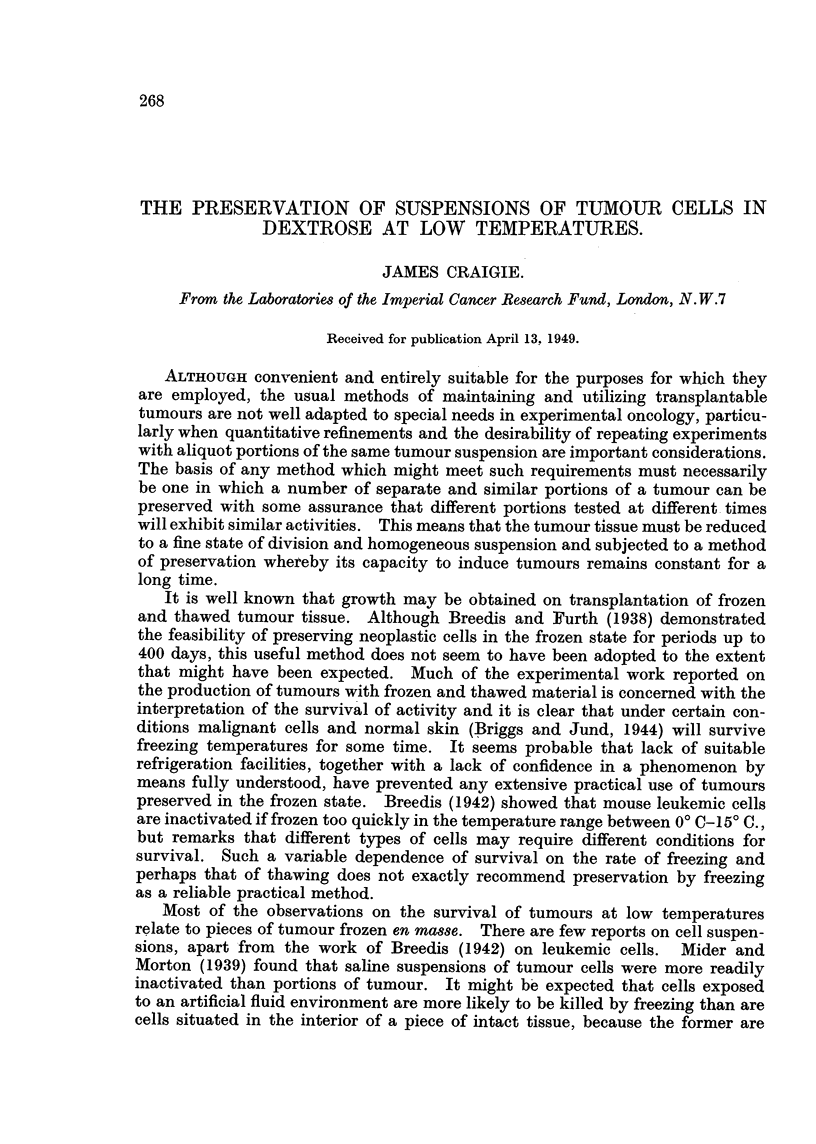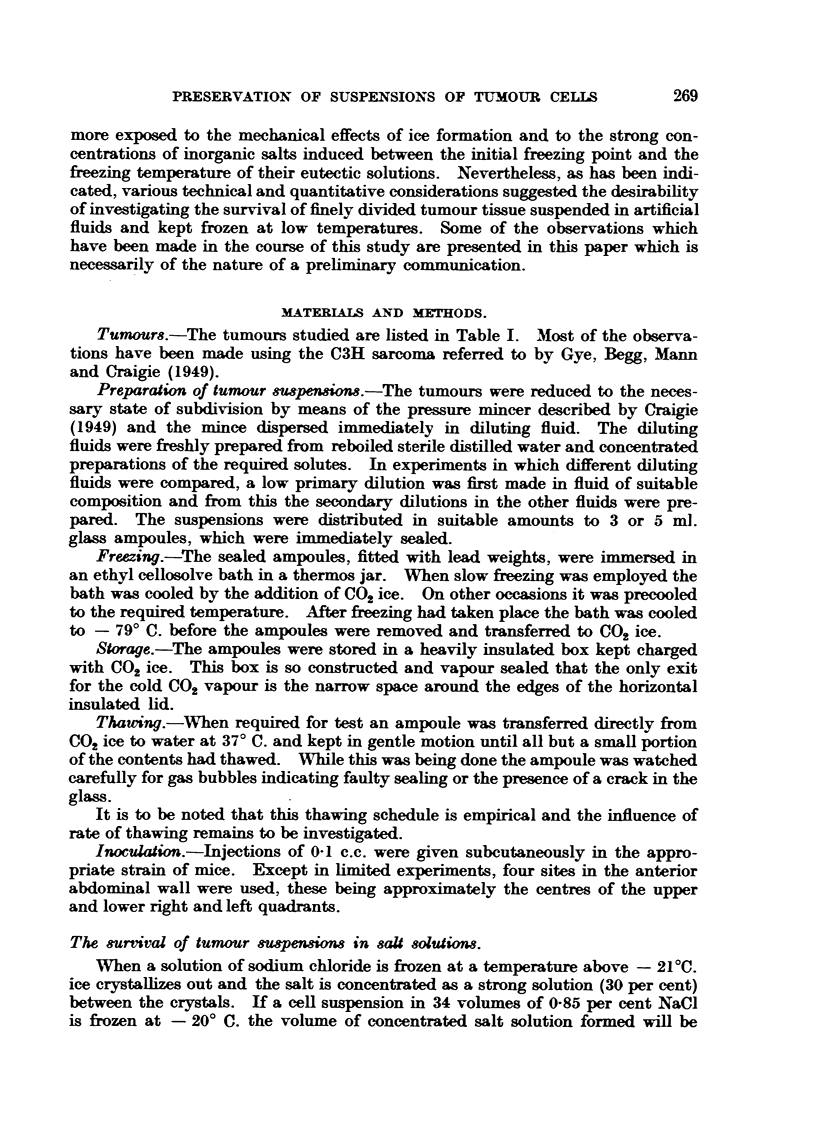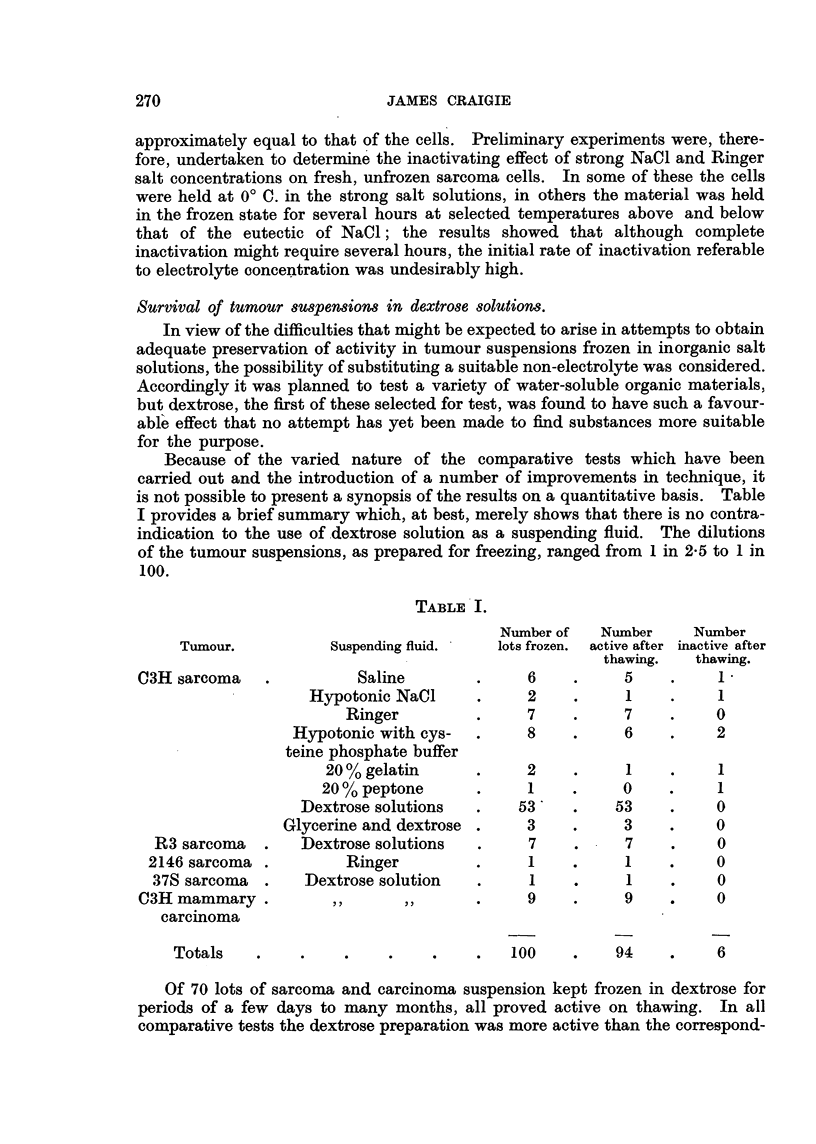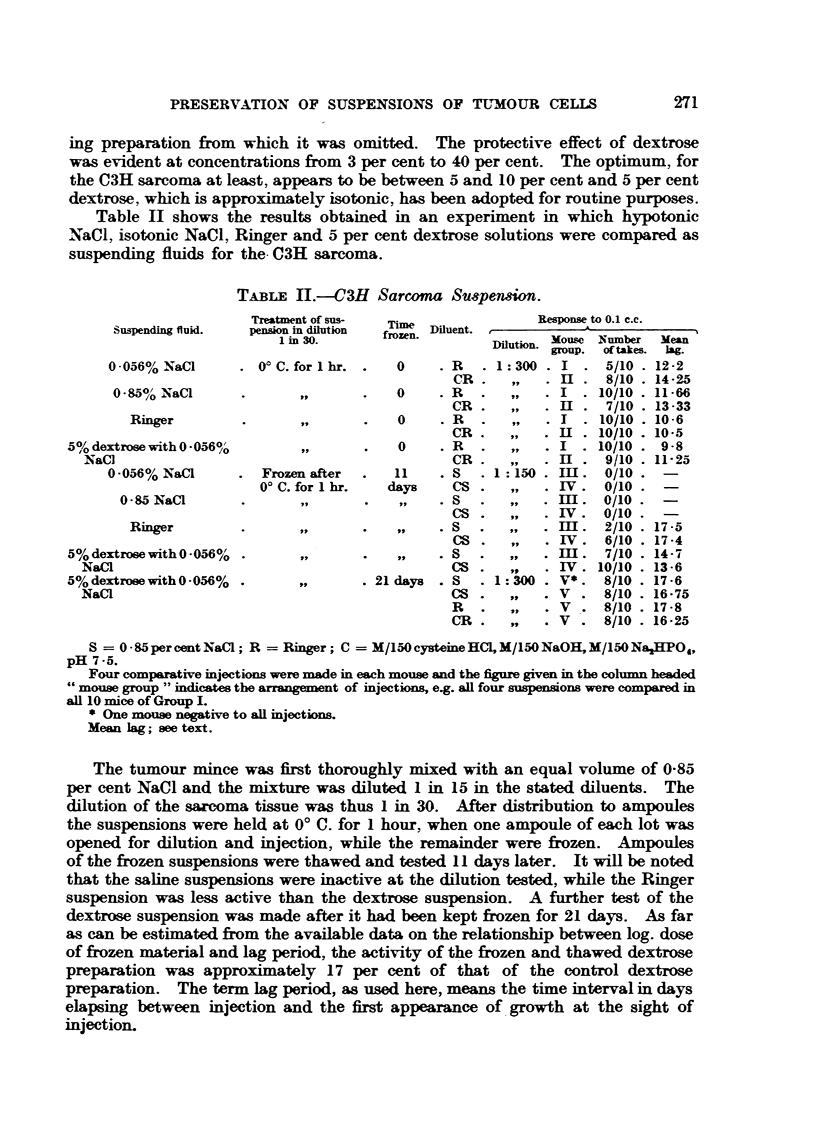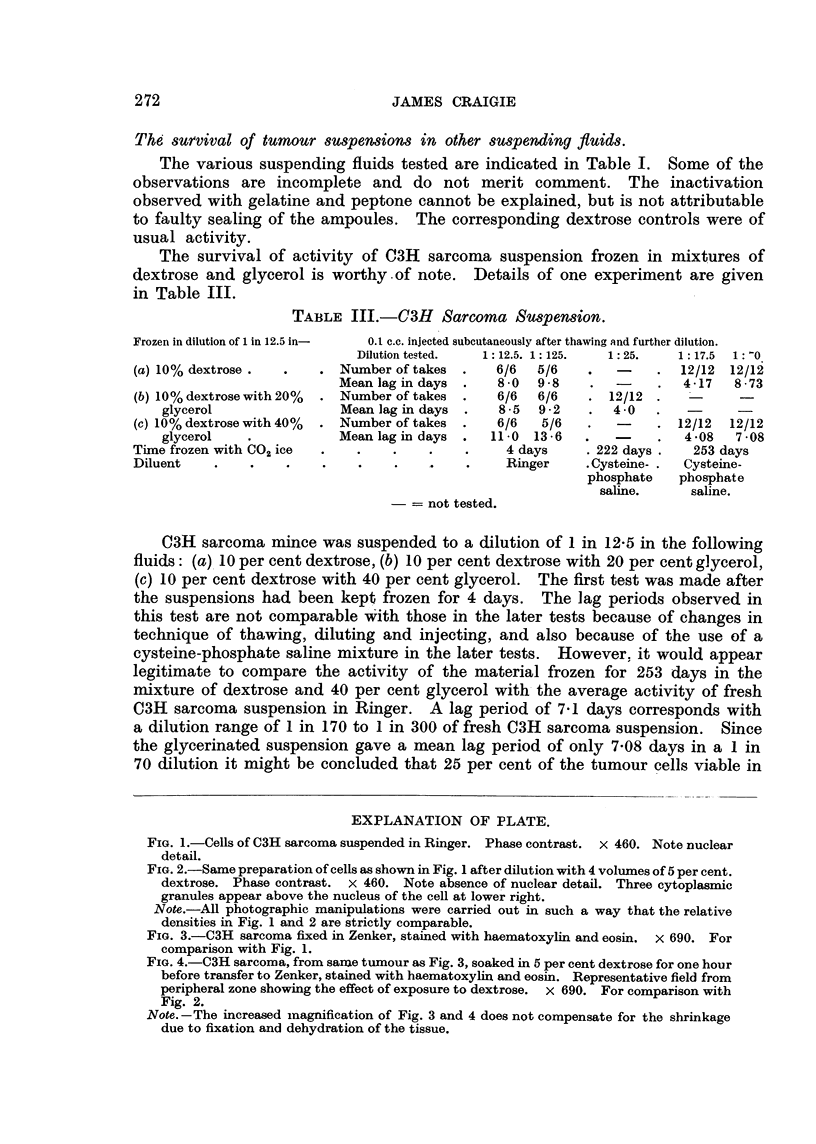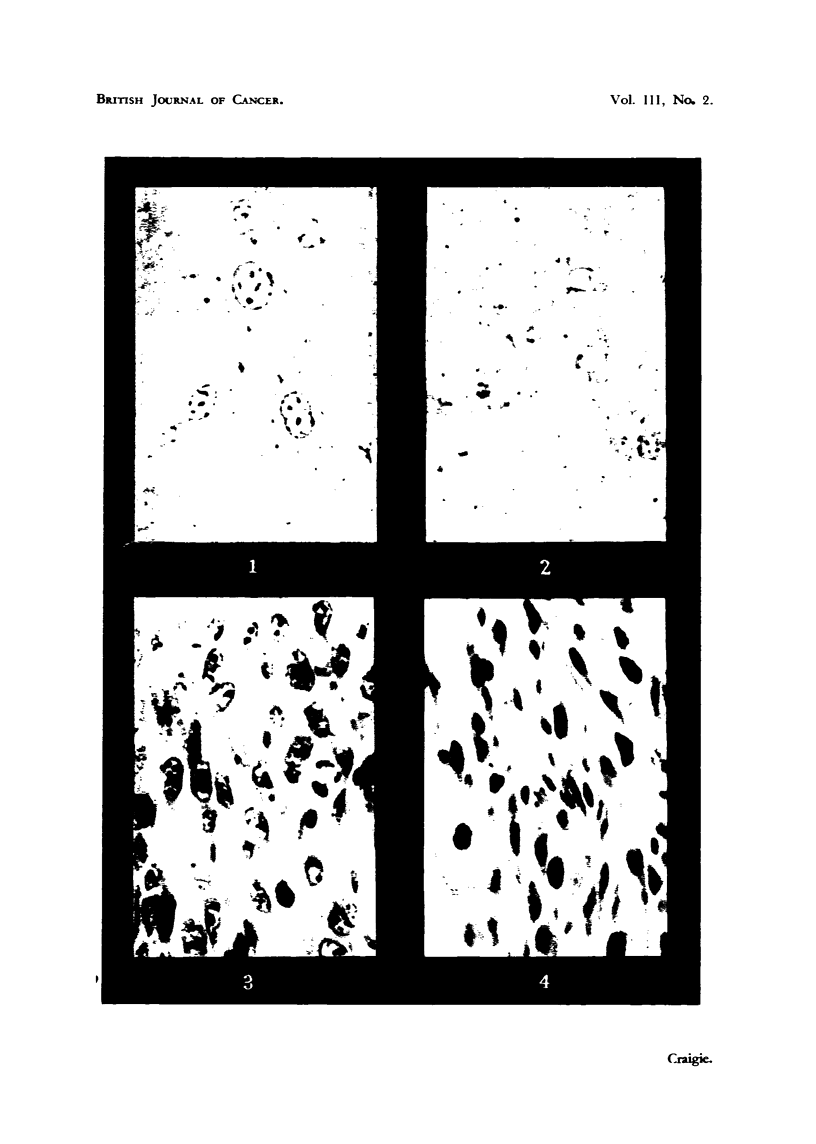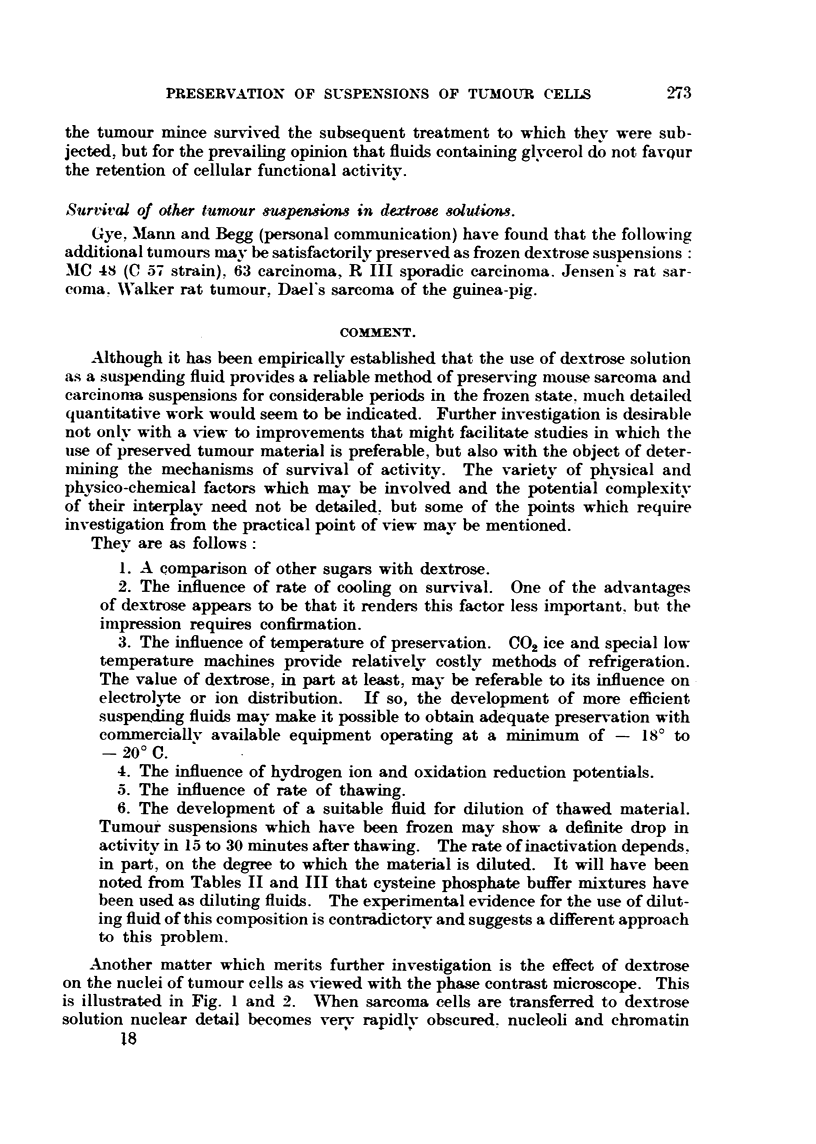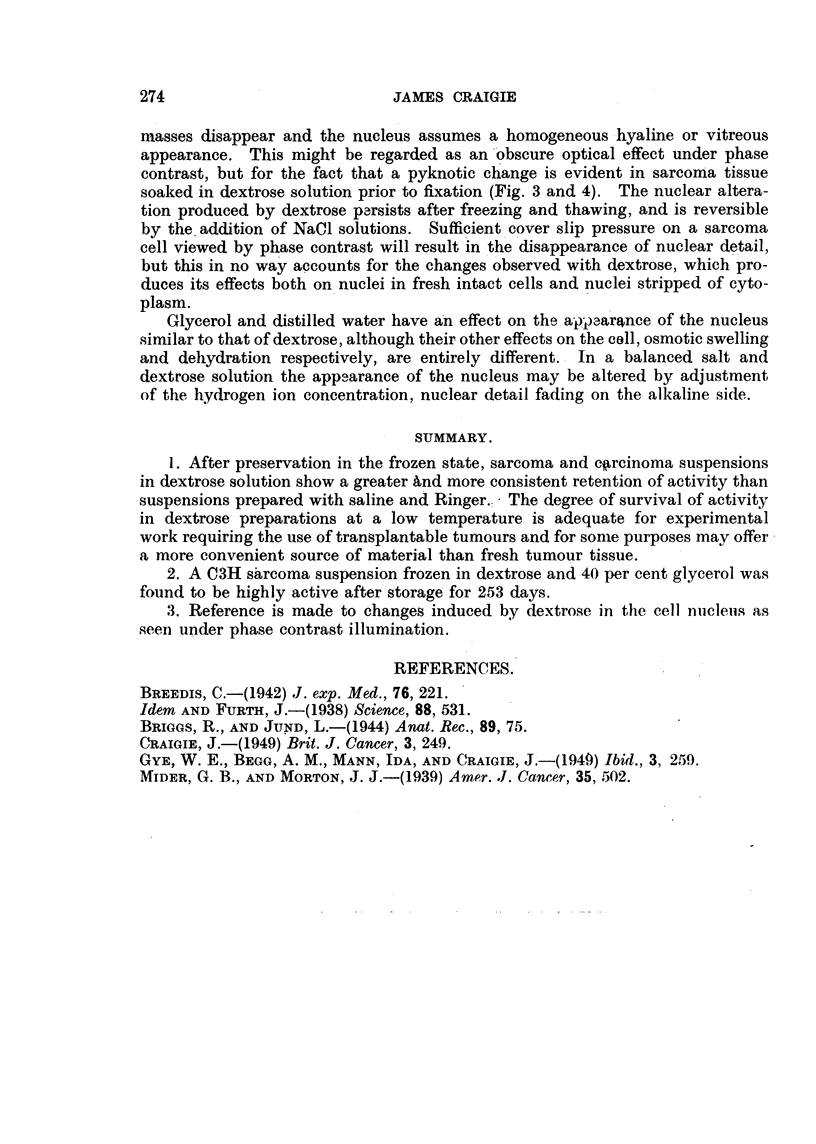# The Preservation of Suspensions of Tumour Cells in Dextrose at Low Temperatures

**DOI:** 10.1038/bjc.1949.30

**Published:** 1949-06

**Authors:** James Craigie

## Abstract

**Images:**


### THE PRESERVATION OF SUSPENSIONS OF TUMOUR CELLS IN DEXTROSE AT LOW TEMPERATURES.

JAMES CRAIGIE.

*From the Laboratories of the Imperial Cancer Research Fund, London, N.W.7*

Received for publication April 13, 1949.

ALTHOUGH convenient and entirely suitable for the purposes for which they are employed, the usual methods of maintaining and utilizing transplantable tumours are not well adapted to special needs in experimental oncology, particularly when quantitative refinements and the desirability of repeating experiments with aliquot portions of the same tumour suspension are important considerations. The basis of any method which might meet such requirements must necessarily be one in which a number of separate and similar portions of a tumour can be preserved with some assurance that different portions tested at different times will exhibit similar activities. This means that the tumour tissue must be reduced to a fine state of division and homogeneous suspension and subjected to a method of preservation whereby its capacity to induce tumours remains constant for a long time.

It is well known that growth may be obtained on transplantation of frozen and thawed tumour tissue. Although Breedis and Furth (1938) demonstrated the feasibility of preserving neoplastic cells in the frozen state for periods up to 400 days, this useful method does not seem to have been adopted to the extent that might have been expected. Much of the experimental work reported on the production of tumours with frozen and thawed material is concerned with the interpretation of the survival of activity and it is clear that under certain conditions malignant cells and normal skin (Briggs and Jund, 1944) will survive freezing temperatures for some time. It seems probable that lack of suitable refrigeration facilities, together with a lack of confidence in a phenomenon by means fully understood, have prevented any extensive practical use of tumours preserved in the frozen state. Breedis (1942) showed that mouse leukemic cells are inactivated if frozen too quickly in the temperature range between 0° C–15° C., but remarks that different types of cells may require different conditions for survival. Such a variable dependence of survival on the rate of freezing and perhaps that of thawing does not exactly recommend preservation by freezing as a reliable practical method.

Most of the observations on the survival of tumours at low temperatures relate to pieces of tumour frozen *en masse*. There are few reports on cell suspensions, apart from the work of Breedis (1942) on leukemic cells. Mider and Morton (1939) found that saline suspensions of tumour cells were more readily inactivated than portions of tumour. It might be expected that cells exposed to an artificial fluid environment are more likely to be killed by freezing than are cells situated in the interior of a piece of intact tissue, because the former are more exposed to the mechanical effects of ice formation and to the strong concentrations of inorganic salts induced between the initial freezing point and the freezing temperature of their eutectic solutions. Nevertheless, as has been indicated, various technical and quantitative considerations suggested the desirability of investigating the survival of finely divided tumour tissue suspended in artificial fluids and kept frozen at low temperatures. Some of the observations which have been made in the course of this study are presented in this paper which is necessarily of the nature of a preliminary communication.

### MATERIALS AND METHODS.

*Tumours.*—The tumours studied are listed in Table I. Most of the observations have been made using the C3H sarcoma referred to by Gye, Begg, Mann and Craigie (1949).

*Preparation of tumour suspensions.*—The tumours were reduced to the necessary state of subdivision by means of the pressure mincer described by Craigie (1949) and the mince dispersed immediately in diluting fluid. The diluting fluids were freshly prepared from reboiled sterile distilled water and concentrated preparations of the required solutes. In experiments in which different diluting fluids were compared, a low primary dilution was first made in fluid of suitable composition and from this the secondary dilutions in the other fluids were prepared. The suspensions were distributed in suitable amounts to 3 or 5 ml. glass ampoules, which were immediately sealed.

*Freezing.*—The sealed ampoules, fitted with lead weights, were immersed in an ethyl cellosolve bath in a thermos jar. When slow freezing was employed the bath was cooled by the addition of $CO_2$ ice. On other occasions it was precooled to the required temperature. After freezing had taken place the bath was cooled to $-79°$ C. before the ampoules were removed and transferred to $CO_2$ ice.

*Storage.*—The ampoules were stored in a heavily insulated box kept charged with $CO_2$ ice. This box is so constructed and vapour sealed that the only exit for the cold $CO_2$ vapour is the narrow space around the edges of the horizontal insulated lid.

*Thawing.*—When required for test an ampoule was transferred directly from $CO_2$ ice to water at 37° C. and kept in gentle motion until all but a small portion of the contents had thawed. While this was being done the ampoule was watched carefully for gas bubbles indicating faulty sealing or the presence of a crack in the glass.

It is to be noted that this thawing schedule is empirical and the influence of rate of thawing remains to be investigated.

*Inoculation.*—Injections of 0·1 c.c. were given subcutaneously in the appropriate strain of mice. Except in limited experiments, four sites in the anterior abdominal wall were used, these being approximately the centres of the upper and lower right and left quadrants.

### *The survival of tumour suspensions in salt solutions.*

When a solution of sodium chloride is frozen at a temperature above $-21°$C. ice crystallizes out and the salt is concentrated as a strong solution (30 per cent) between the crystals. If a cell suspension in 34 volumes of 0·85 per cent NaCl is frozen at $-20°$ C. the volume of concentrated salt solution formed will be approximately equal to that of the cells. Preliminary experiments were, therefore, undertaken to determine the inactivating effect of strong NaCl and Ringer salt concentrations on fresh, unfrozen sarcoma cells. In some of these the cells were held at 0° C. in the strong salt solutions, in others the material was held in the frozen state for several hours at selected temperatures above and below that of the eutectic of NaCl; the results showed that although complete inactivation might require several hours, the initial rate of inactivation referable to electrolyte concentration was undesirably high.

*Survival of tumour suspensions in dextrose solutions.*

In view of the difficulties that might be expected to arise in attempts to obtain adequate preservation of activity in tumour suspensions frozen in inorganic salt solutions, the possibility of substituting a suitable non-electrolyte was considered. Accordingly it was planned to test a variety of water-soluble organic materials, but dextrose, the first of these selected for test, was found to have such a favourable effect that no attempt has yet been made to find substances more suitable for the purpose.

Because of the varied nature of the comparative tests which have been carried out and the introduction of a number of improvements in technique, it is not possible to present a synopsis of the results on a quantitative basis. Table I provides a brief summary which, at best, merely shows that there is no contra-indication to the use of dextrose solution as a suspending fluid. The dilutions of the tumour suspensions, as prepared for freezing, ranged from 1 in 2·5 to 1 in 100.

TABLE I.

| Tumour. | Suspending fluid. | Number of lots frozen. | Number active after thawing. | Number inactive after thawing. |
|---|---|---|---|---|
| C3H sarcoma | Saline | 6 | 5 | 1 |
| | Hypotonic NaCl | 2 | 1 | 1 |
| | Ringer | 7 | 7 | 0 |
| | Hypotonic with cysteine phosphate buffer | 8 | 6 | 2 |
| | 20 % gelatin | 2 | 1 | 1 |
| | 20 % peptone | 1 | 0 | 1 |
| | Dextrose solutions | 53 | 53 | 0 |
| | Glycerine and dextrose | 3 | 3 | 0 |
| R3 sarcoma | Dextrose solutions | 7 | 7 | 0 |
| 2146 sarcoma | Ringer | 1 | 1 | 0 |
| 37S sarcoma | Dextrose solution | 1 | 1 | 0 |
| C3H mammary carcinoma | ,, ,, | 9 | 9 | 0 |
| Totals | | 100 | 94 | 6 |

Of 70 lots of sarcoma and carcinoma suspension kept frozen in dextrose for periods of a few days to many months, all proved active on thawing. In all comparative tests the dextrose preparation was more active than the corresponding preparation from which it was omitted. The protective effect of dextrose was evident at concentrations from 3 per cent to 40 per cent. The optimum, for the C3H sarcoma at least, appears to be between 5 and 10 per cent and 5 per cent dextrose, which is approximately isotonic, has been adopted for routine purposes.

Table II shows the results obtained in an experiment in which hypotonic NaCl, isotonic NaCl, Ringer and 5 per cent dextrose solutions were compared as suspending fluids for the C3H sarcoma.

TABLE II.—*C3H Sarcoma Suspension.*

| Suspending fluid. | Treatment of suspension in dilution 1 in 30. | Time frozen. | Diluent. | Response to 0.1 c.c. | | | |
|---|---|---|---|---|---|---|---|
| | | | | Dilution. | Mouse group. | Number of takes. | Mean lag. |
| 0·056% NaCl | 0° C. for 1 hr. | 0 | R | 1 : 300 | I | 5/10 | 12·2 |
| | | | CR | „ | II | 8/10 | 14·25 |
| 0·85% NaCl | „ | 0 | R | „ | I | 10/10 | 11·66 |
| | | | CR | „ | II | 7/10 | 13·33 |
| Ringer | „ | 0 | R | „ | I | 10/10 | 10·6 |
| | | | CR | „ | II | 10/10 | 10·5 |
| 5% dextrose with 0·056% NaCl | „ | 0 | R | „ | I | 10/10 | 9·8 |
| | | | CR | „ | II | 9/10 | 11·25 |
| 0·056% NaCl | Frozen after 0° C. for 1 hr. | 11 days | S | 1 : 150 | III | 0/10 | — |
| | | | CS | „ | IV | 0/10 | — |
| 0·85 NaCl | „ | „ | S | „ | III | 0/10 | — |
| | | | CS | „ | IV | 0/10 | — |
| Ringer | „ | „ | S | „ | III | 2/10 | 17·5 |
| | | | CS | „ | IV | 6/10 | 17·4 |
| 5% dextrose with 0·056% NaCl | „ | „ | S | „ | III | 7/10 | 14·7 |
| | | | CS | „ | IV | 10/10 | 13·6 |
| 5% dextrose with 0·056% NaCl | „ | 21 days | S | 1 : 300 | V* | 8/10 | 17·6 |
| | | | CS | „ | V | 8/10 | 16·75 |
| | | | R | „ | V | 8/10 | 17·8 |
| | | | CR | „ | V | 8/10 | 16·25 |

S = 0·85 per cent NaCl ; R = Ringer ; C = M/150 cysteine HCl, M/150 NaOH, M/150 $Na_2HPO_4$, pH 7·5.

Four comparative injections were made in each mouse and the figure given in the column headed " mouse group " indicates the arrangement of injections, e.g. all four suspensions were compared in all 10 mice of Group I.

* One mouse negative to all injections.

Mean lag; see text.

The tumour mince was first thoroughly mixed with an equal volume of 0·85 per cent NaCl and the mixture was diluted 1 in 15 in the stated diluents. The dilution of the sarcoma tissue was thus 1 in 30. After distribution to ampoules the suspensions were held at 0° C. for 1 hour, when one ampoule of each lot was opened for dilution and injection, while the remainder were frozen. Ampoules of the frozen suspensions were thawed and tested 11 days later. It will be noted that the saline suspensions were inactive at the dilution tested, while the Ringer suspension was less active than the dextrose suspension. A further test of the dextrose suspension was made after it had been kept frozen for 21 days. As far as can be estimated from the available data on the relationship between log. dose of frozen material and lag period, the activity of the frozen and thawed dextrose preparation was approximately 17 per cent of that of the control dextrose preparation. The term lag period, as used here, means the time interval in days elapsing between injection and the first appearance of growth at the sight of injection.

*The survival of tumour suspensions in other suspending fluids.*

The various suspending fluids tested are indicated in Table I. Some of the observations are incomplete and do not merit comment. The inactivation observed with gelatine and peptone cannot be explained, but is not attributable to faulty sealing of the ampoules. The corresponding dextrose controls were of usual activity.

The survival of activity of C3H sarcoma suspension frozen in mixtures of dextrose and glycerol is worthy of note. Details of one experiment are given in Table III.

TABLE III.—*C3H Sarcoma Suspension.*

| Frozen in dilution of 1 in 12.5 in— | 0.1 c.c. injected subcutaneously after thawing and further dilution. | | | | | |
|---|---|---|---|---|---|---|
| | Dilution tested. | 1 : 12.5. | 1 : 125. | 1 : 25. | 1 : 17.5 | 1 : 70 |
| (*a*) 10% dextrose | Number of takes | 6/6 | 5/6 | — | 12/12 | 12/12 |
| | Mean lag in days | 8·0 | 9·8 | — | 4·17 | 8·73 |
| (*b*) 10% dextrose with 20% glycerol | Number of takes | 6/6 | 6/6 | 12/12 | — | — |
| | Mean lag in days | 8·5 | 9·2 | 4·0 | — | — |
| (*c*) 10% dextrose with 40% glycerol | Number of takes | 6/6 | 5/6 | — | 12/12 | 12/12 |
| | Mean lag in days | 11·0 | 13·6 | — | 4·08 | 7·08 |
| Time frozen with $CO_2$ ice | | 4 days | | 222 days | 253 days | |
| Diluent | | Ringer | | Cysteine-phosphate saline. | Cysteine-phosphate saline. | |

— = not tested.

C3H sarcoma mince was suspended to a dilution of 1 in 12·5 in the following fluids: (*a*) 10 per cent dextrose, (*b*) 10 per cent dextrose with 20 per cent glycerol, (*c*) 10 per cent dextrose with 40 per cent glycerol. The first test was made after the suspensions had been kept frozen for 4 days. The lag periods observed in this test are not comparable with those in the later tests because of changes in technique of thawing, diluting and injecting, and also because of the use of a cysteine-phosphate saline mixture in the later tests. However, it would appear legitimate to compare the activity of the material frozen for 253 days in the mixture of dextrose and 40 per cent glycerol with the average activity of fresh C3H sarcoma suspension in Ringer. A lag period of 7·1 days corresponds with a dilution range of 1 in 170 to 1 in 300 of fresh C3H sarcoma suspension. Since the glycerinated suspension gave a mean lag period of only 7·08 days in a 1 in 70 dilution it might be concluded that 25 per cent of the tumour cells viable in

---

EXPLANATION OF PLATE.

FIG. 1.—Cells of C3H sarcoma suspended in Ringer. Phase contrast. × 460. Note nuclear detail.

FIG. 2.—Same preparation of cells as shown in Fig. 1 after dilution with 4 volumes of 5 per cent. dextrose. Phase contrast. × 460. Note absence of nuclear detail. Three cytoplasmic granules appear above the nucleus of the cell at lower right.

*Note.*—All photographic manipulations were carried out in such a way that the relative densities in Fig. 1 and 2 are strictly comparable.

FIG. 3.—C3H sarcoma fixed in Zenker, stained with haematoxylin and eosin. × 690. For comparison with Fig. 1.

FIG. 4.—C3H sarcoma, from same tumour as Fig. 3, soaked in 5 per cent dextrose for one hour before transfer to Zenker, stained with haematoxylin and eosin. Representative field from peripheral zone showing the effect of exposure to dextrose. × 690. For comparison with Fig. 2.

*Note.*—The increased magnification of Fig. 3 and 4 does not compensate for the shrinkage due to fixation and dehydration of the tissue.

Craigie.

the tumour mince survived the subsequent treatment to which they were subjected, but for the prevailing opinion that fluids containing glycerol do not favour the retention of cellular functional activity.

*Survival of other tumour suspensions in dextrose solutions.*

Gye, Mann and Begg (personal communication) have found that the following additional tumours may be satisfactorily preserved as frozen dextrose suspensions : MC 48 (C 57 strain), 63 carcinoma, R III sporadic carcinoma. Jensen's rat sarcoma. Walker rat tumour, Dael's sarcoma of the guinea-pig.

### COMMENT.

Although it has been empirically established that the use of dextrose solution as a suspending fluid provides a reliable method of preserving mouse sarcoma and carcinoma suspensions for considerable periods in the frozen state, much detailed quantitative work would seem to be indicated. Further investigation is desirable not only with a view to improvements that might facilitate studies in which the use of preserved tumour material is preferable, but also with the object of determining the mechanisms of survival of activity. The variety of physical and physico-chemical factors which may be involved and the potential complexity of their interplay need not be detailed, but some of the points which require investigation from the practical point of view may be mentioned. They are as follows :

1. A comparison of other sugars with dextrose.
2. The influence of rate of cooling on survival. One of the advantages of dextrose appears to be that it renders this factor less important, but the impression requires confirmation.
3. The influence of temperature of preservation. $CO_2$ ice and special low temperature machines provide relatively costly methods of refrigeration. The value of dextrose, in part at least, may be referable to its influence on electrolyte or ion distribution. If so, the development of more efficient suspending fluids may make it possible to obtain adequate preservation with commercially available equipment operating at a minimum of $-18°$ to $-20°$ C.
4. The influence of hydrogen ion and oxidation reduction potentials.
5. The influence of rate of thawing.
6. The development of a suitable fluid for dilution of thawed material. Tumour suspensions which have been frozen may show a definite drop in activity in 15 to 30 minutes after thawing. The rate of inactivation depends, in part, on the degree to which the material is diluted. It will have been noted from Tables II and III that cysteine phosphate buffer mixtures have been used as diluting fluids. The experimental evidence for the use of diluting fluid of this composition is contradictory and suggests a different approach to this problem.

Another matter which merits further investigation is the effect of dextrose on the nuclei of tumour cells as viewed with the phase contrast microscope. This is illustrated in Fig. 1 and 2. When sarcoma cells are transferred to dextrose solution nuclear detail becomes very rapidly obscured, nucleoli and chromatin masses disappear and the nucleus assumes a homogeneous hyaline or vitreous appearance. This might be regarded as an obscure optical effect under phase contrast, but for the fact that a pyknotic change is evident in sarcoma tissue soaked in dextrose solution prior to fixation (Fig. 3 and 4). The nuclear alteration produced by dextrose persists after freezing and thawing, and is reversible by the addition of NaCl solutions. Sufficient cover slip pressure on a sarcoma cell viewed by phase contrast will result in the disappearance of nuclear detail, but this in no way accounts for the changes observed with dextrose, which produces its effects both on nuclei in fresh intact cells and nuclei stripped of cytoplasm.

Glycerol and distilled water have an effect on the appearance of the nucleus similar to that of dextrose, although their other effects on the cell, osmotic swelling and dehydration respectively, are entirely different. In a balanced salt and dextrose solution the appearance of the nucleus may be altered by adjustment of the hydrogen ion concentration, nuclear detail fading on the alkaline side.

### SUMMARY.

1. After preservation in the frozen state, sarcoma and carcinoma suspensions in dextrose solution show a greater and more consistent retention of activity than suspensions prepared with saline and Ringer. The degree of survival of activity in dextrose preparations at a low temperature is adequate for experimental work requiring the use of transplantable tumours and for some purposes may offer a more convenient source of material than fresh tumour tissue.

2. A C3H sarcoma suspension frozen in dextrose and 40 per cent glycerol was found to be highly active after storage for 253 days.

3. Reference is made to changes induced by dextrose in the cell nucleus as seen under phase contrast illumination.

### REFERENCES.

BREEDIS, C.—(1942) *J. exp. Med.*, **76**, 221.
*Idem* AND FURTH, J.—(1938) *Science*, **88**, 531.
BRIGGS, R., AND JUND, L.—(1944) *Anat. Rec.*, **89**, 75.
CRAIGIE, J.—(1949) *Brit. J. Cancer*, **3**, 249.
GYE, W. E., BEGG, A. M., MANN, IDA, AND CRAIGIE, J.—(1949) *Ibid.*, **3**, 259.
MIDER, G. B., AND MORTON, J. J.—(1939) *Amer. J. Cancer*, **35**, 502.